# Toward steering the motion of surface rolling molecular machines by straining graphene substrate

**DOI:** 10.1038/s41598-023-48214-1

**Published:** 2023-11-27

**Authors:** Mehran Vaezi, Hossein Nejat Pishkenari

**Affiliations:** 1 Center for Nanoscience and Nanotechnology, Institute for Convergence Science & Technology , Sharif University of Technology, Tehran, Iran; 2 Nano Robotics Laboratory, Mechanical Engineering Department, Sharif University of Technology, Tehran, Iran

**Keywords:** Nanoscience and technology, Graphene, Nanoscale devices, Nanoscale materials

## Abstract

The surface rolling molecular machines are proposed to perform tasks and carrying molecular payloads on the substrates. As a result, controlling the surface motion of these molecular machines is of interest for the design of nano-transportation systems. In this study, we evaluate the motion of the nanocar on the graphene nanoribbons with strain gradient, through the molecular dynamics (MD) simulations, and theoretical relations. The nanocar indicates directed motion from the maximum strained part of the graphene to the unstrained end of the substrate. The strain gradient induced driving force and diffusion coefficients of nanocars are analyzed from the simulation and theoretical points of view. To obtain the optimum directed motion of nanocar, we consider the effects of temperature, strain average, and magnitude of strain gradient on the directionality of the motion. Moreover, the mechanism of the motion of nanocar is studied by evaluating the direction of the nanocar’s chassis and the rotation of wheels around the axles. Ultimately, the programmable motion of nanocar is shown by adjusting the strain gradient of graphene substrate.

The first attempts for the fabrication of nanomachines were started by the investigations of Sauvage et al.^1^, where two molecular rings were interlocked mechanically (named as catenane structure). The idea of synthesis of molecular machines was followed by Fraser Stoddart and his colleagues^2^. In 1991, the group reported the synthesis of a molecular structure which is called as “molecular shuttle”^3^. In this molecular architecture, a macrocycle is mechanically interlocked in a dumbbell-shaped molecular chain. The macrocycle is able to switch between two stable stations on the molecular chain by changing the temperature. Later in 2016, Jean-Pierre Sauvage, Fraser Stoddart and Ben Feringa jointly received the Nobel Prize in Chemistry for their efforts in developing the first molecular machines^4^. In 2006, James tour and his colleagues synthesized the first surface rolling molecular machines named as “Nanocar”^5^. These nanomachines are developed to perform special tasks, such as transporting materials and energy on the surface. Similar to the conventional vehicles, the nanocars consist of molecular wheels, axles and chassis^6,7^. The fullerene molecule is one of the most popular candidates for use as the wheels of nanocars^7–9^, due to the spherical and symmetry of the molecule. In this study, we examine the motion of a nanotruck, which is a fullerene-based nanocar with rigid chassis.

Since the nanocars have shown the ability of carrying molecular cargoes, controlling the surface motion of these structures has received attentions in the previous studies^10–12^. Nemati et al.^13,14^, introduced the atomic modification of the surface as a method to direct the movements of nanocars. The restricted motion of fullerene-based nanocars has been reported on the top side of the atomic steps of the gold substrate^13^. The limited surface motions of C60 and nanocars are also observed, when the molecules are in the atomic vacancies of the gold substrate. Using hybrid substrates is another technique to guide the surface mobility of the nanocars^15^. Nemati and his colleagues^16^ simulated the motion of nanocars on the silver substrates with gold region impurities. According to this investigation, the nanocars prefer to move on gold areas, due to the lower potential energy of the nanomachines on gold atoms.

Other methods are proposed to steer the surface motion of nanomachines, such as employing external electric field on the nanocar^17^, and temperature gradient on the substrate^18^. The electric field on scanning tunneling microscope (STM) is commonly used in experiments to guide the motion of nanocars on the surface^19^. This method has also been utilized in the first^20^ and second^21^ nanocars race (held in 2017 and 2022), to propel the nanovehicles on the gold surface. The temperature gradient of the substrate was shown to be effective in controlling the surface motion of C60-based nanocars^18^ and other molecules^22^. The free energy of nanomachines decreases as they move toward the region of the substrate which has lower temperature^23^. Galvão et al.^24^ demonstrated that, the curvature gradient of the substrate is able to provide mechanical stimuli to direct the motion. According to this study, the spiral-shaped substrates create gradients of bending energy which leads to the driving force on the nanomaterial located on the surface. Previously, the surface energy gradient was also employed to drive nanotube-based oscillators^25^.

Directed surface motion of molecules was obtained by applying strain gradient on the substrate^26^. Huang et al.^27^ reported the directional transport of carbon nanotube (CNT) and graphene nanoflake on the graphene substrates with strain gradient. The nanoflake and CNT are capable of carrying payloads such as metal nanoparticles to the less strained part of the substrate^27^. The strain gradient of the substrate has been employed to control the rotational motions on the surface, as well. According to the computational study conducted by Khan et al.^28^, the graphene nanoflake hinged to the graphene substrate starts to rotate to the unstrained area of the surface as the substrate is locally indented. It is concluded that, rotational motion of nanoflake is controllable by indenting different points of the graphene layer. Mofidi et al.^29^ demonstrated the rectilinear motion of C60 molecule on the graphene layer with uniaxial strain. The analysis of the potential energy reveals that, uniaxial strain of the substrate leads to the straight pathways on the surface, in which the molecule experiences lower potential energy. As a result, the fullerene shows rectilinear motion on these pathways which are energetically more favorable.

Using molecular dynamics simulations and analytical model, Huang et al.^30^ showed that the strain field can be used as a real-time controlling method for nano-actuator system consists of double-walled carbon nanotube and graphene substrate. According to this study, rotational speed of outer tube is controllable by altering the strain field through the change of the C-C bond length and inter-tube distance. Huang et al. continued their investigation, by considering the effect of axial strain on the linear motion of the double walled carbon nanotube thermal actuator^31^. Based on the results of the mentioned study, the axial strain can be employed to steer the linear movements of the double walled carbon nanotube thermal actuator.

In the present investigation, using molecular dynamics simulations, we study the directed motion of the nanocar on the graphene layer with strain gradient. The effect of the magnitude of strain gradient is evaluated by choosing the strain gradient range of 5 to 20 percent. The driving force on the nanocars and their diffusion coefficients are considered from the theoretical aspects, as well as the numerical simulations. We study the influence of the strain gradient of substrate on the nanocar’s mechanism of the motion (wheel rolling and sliding mechanisms). The directional motion of the nanocar is examined at various temperatures from 200 to 500 K. To obtain a targeted manipulation, we try to control the surface motion of the nanocar by changing strain gradient of the graphene layer.

## Computational method

**Figure 1:**
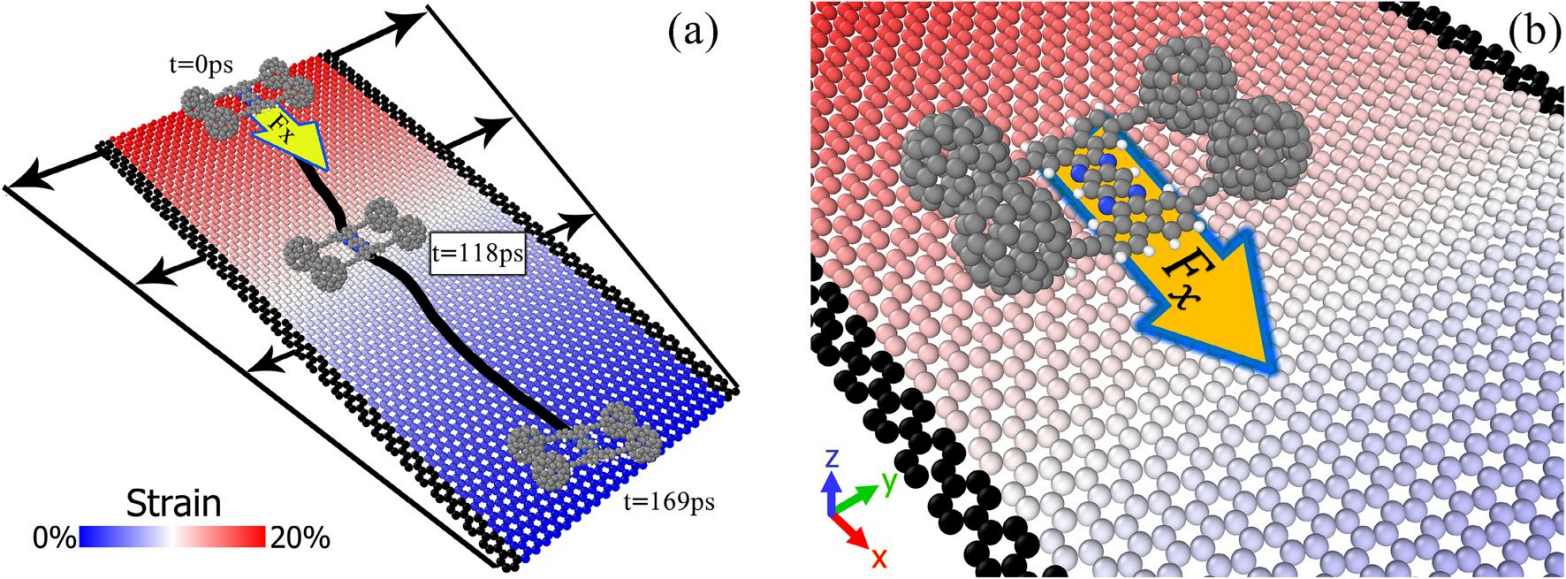
Steered motion of nanotruck on the graphene layer with strain gradient. (**a**) The color-bar represents the strain variation on the substrate. (**b**) The coordinate system is also demonstrated in the magnified figure.

The motion of the nanomachines was simulated on the graphene layer by molecular dynamics method. This method is able to predict the dynamics of materials at nano-scale and in a short period of time, which is hard to be observed through the experiments^32–34^. A graphene monolayer with the dimensions of 70 Å × 150 Å was considered as the substrate of the nanocar, due to its appropriate mechanical and electrical properties^35–38^. The dimensions of the cubic simulation box are 170 Å × 80 Å × 50 Å in the directions of $x$-, $y$- and $z$-axes, respectively. The center of the simulation box is defined at the origin of the coordinate system. According to Fig. 1, the strain uniformly decreases along the length of the graphene layer. To stretch the substrate, before starting the main simulations, two narrow strips of the graphene layer (black atoms in Fig. 1) were displaced with the velocity of 1 Å/ps and in the direction of y-axis. To maintain the strain gradient, these narrow strips were considered fixed during the simulations, while other atoms of the substrate are allowed to move.

A special type of nanocar was utilized in different simulations, which is named as “Nanotruck”^39^. The structure of nanomachine consists of four C60 wheels and it has the average dimensions of 29.7Å× 18.9Å. The chemical formula of nanotruck is C_282_H_18_N_4_ and it shows D_2_ symmetry^40^.

The interactions among the graphene atoms were modeled by Tersoff potential^41^. The inner interactions of nanocars atoms were described with Molecular Mechanics (MM3) force field^42^. The mentioned force field is commonly used for the simulation of nanocars^43,44^. “HA” and “NA” atom types are assigned to the hydrogen and nitrogen atoms of nanocar. Two atom types are dedicated to the carbon atoms of nanomachine depending on their hybridizations. For carbon atoms with the hybridization of *sp*^*2*^ and *sp*^*3*^, we considered “CA” type and “C2” atom type was employed to describe *sp* atoms. The bond and angle terms of the force field are of harmonic type, and the general form of dihedral term is demonstrated in Eq. (1).

1 $$E_{dihedral} = \frac{1}{2}K_{1} \left( {1 + \cos \left( \varphi \right)} \right) + \frac{1}{2}K_{2} \left( {1 - \cos \left( {2\varphi } \right)} \right) + \frac{1}{2}K_{3} \left( {1 + \cos \left( {3\varphi } \right)} \right) + \frac{1}{2}K_{4} \left( {1 - \cos \left( {4\varphi } \right)} \right)$$

where dihedral angle is shown by $\varphi$ and ${K}_{1}.{K}_{2}.{K}_{3}$ and ${K}_{4}$ are torsion stiffness. Different parameters of the force field and partial charges of nanocars atoms are similar to our previous investigations^9^. Supporting Information includes input parameters required to conduct LAMMPS simulations. The intermolecular interactions between nanocars and graphene atoms were considered as 6–12 Lennard–Jones (LJ) potential, with the cut-off radius of 12 Å. The following table indicates the LJ parameters employed to describe the nonbonded interactions.

The simulations were performed in canonical (NVT) ensemble at 300 K, which is applied by Nose-Hover^49,50^ thermostat. The potential energy of the system was minimized by conjugate gradient method, to reach the energy tolerance less than ${10}^{-6}$. The velocity Verlet integration method was considered to solve the equations of motion. The time-step of the simulations was adjusted to 1 fs, and different simulations were carried out by LAMMPS package^51^. The simulation data including position, velocity, and the force on the nanocar were captured every 100 steps (i.e., 100 fs) during the path calculations.

## Results and discussion

### Computational and theoretical aspects of directed motion

**Figure 2:**
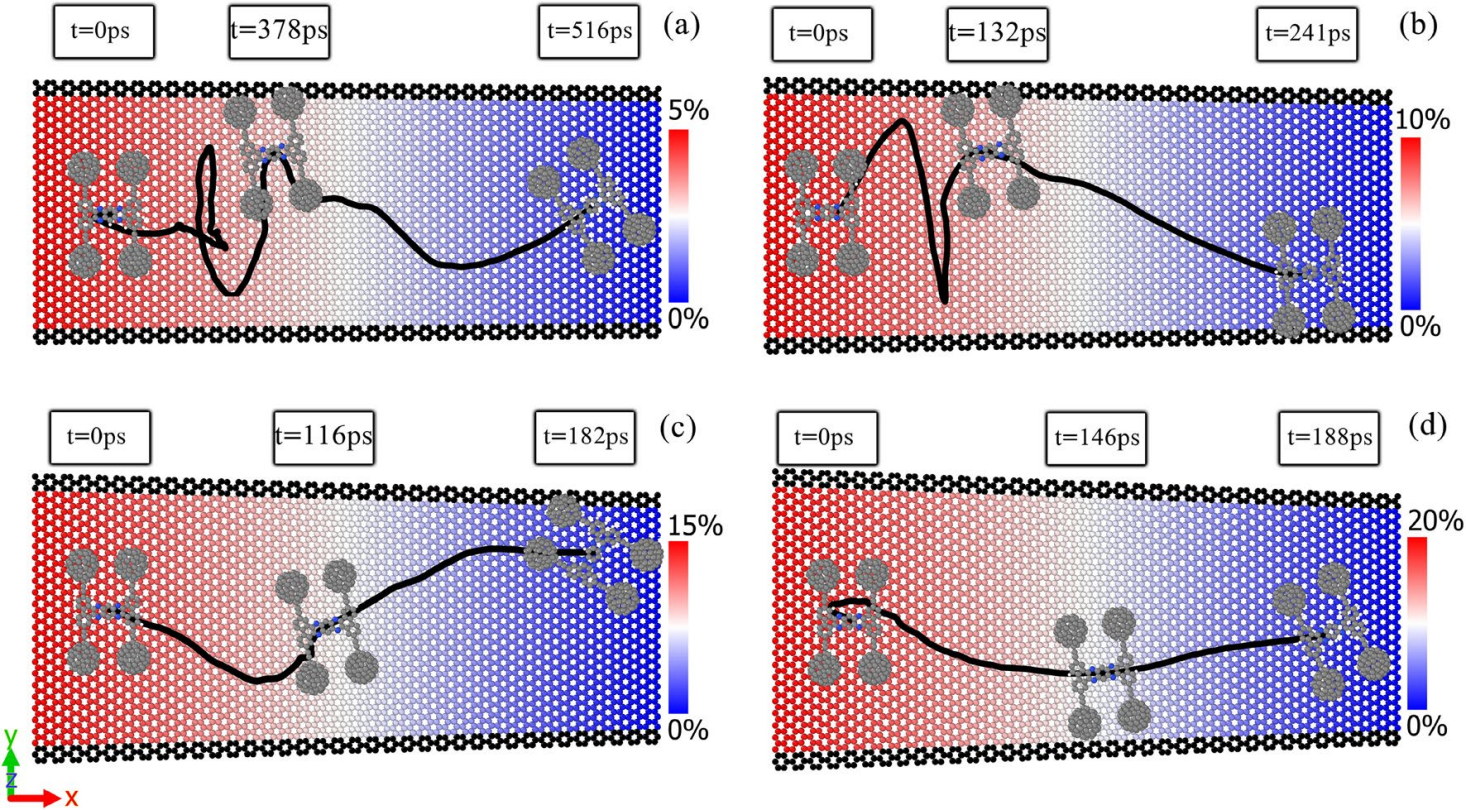
The snapshots of the simulation of nanotruck on the graphene layers with the strain gradient of (**a**) 5%, (**b**) 10%, (**c**) 15%, and (**d**) 20%.

The simulations of the motion of nanotruck on strain gradient surface indicate directed motion of the nanomachine at the temperature of 300 K. According to Fig. 2, the nanocar moves from the left side of the substrate, which has the maximum strain, to the unstrained end of the graphene layer. In the surfaces with the strain gradient, the areal density of atoms decreases as we increase the strain of the substrate. Due to the higher areal density of substrate’s atoms at the unstrained part of the substrate, the nanocar finds stronger interactions and lower potential energy with the surface at this area. As a result, the unstrained side of the substrate is energetically more favorable for the nanomachine. As we observe in Fig. 2d, at higher strain gradient (i.e. 20%), the nanocar travels the length of the graphene layer faster (188 picosecond), and the trajectory of the motion shows more straight path. The increase of the directionality of motion is related to the increase of driving force on the nanocar at higher strain gradient of substrate. We discuss about the theoretical relation between strain gradient of substrate and driving force on the nanocar more clearly at the end of this section.

**Figure 3:**
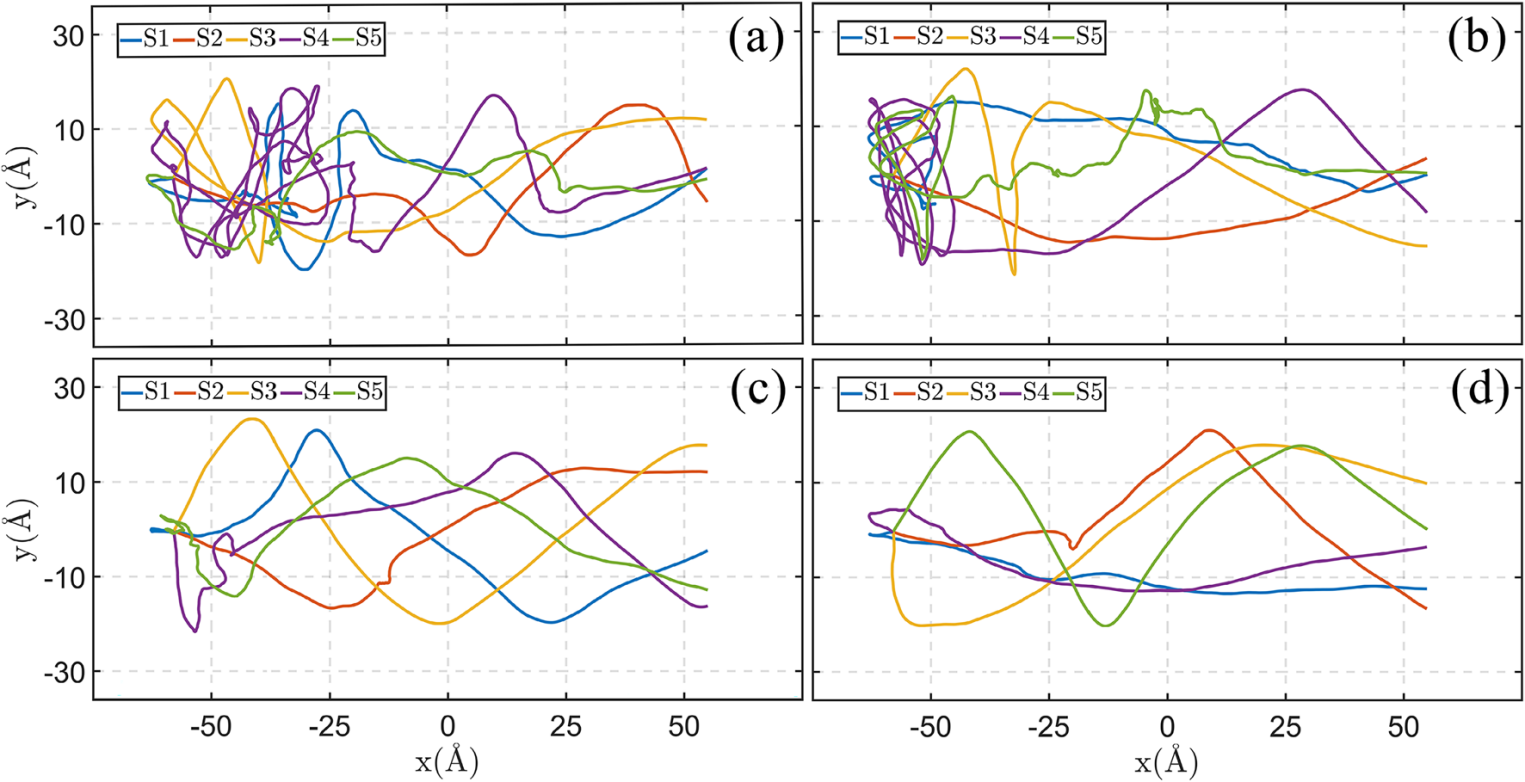
Trajectories of the motion of nanotruck on graphene substrates with strain gradient of (**a**) 5%, (**b**) 10%, (**c**) 15% and (**d**) 20%, at the temperature of 300 K. At each strain gradient, the simulations are repeated with different initial conditions of nanocar (S1–S5), to examine the repeatability of the results.

The repeatability of the obtained results was examined by performing five simulations with different initial conditions of the nanocar. Figure 3a–d demonstrate the trajectories of the motion of nanotruck on the graphene layers with the strain gradient of 5%, 10%, 15% and 20%. At each strain gradient, the nanotruck starts the simulations with five different distributions of initial velocity (different seed numbers). It should be mentioned that, different distributions of initial velocity are corresponding to the same temperature (i.e., 300 K). As the strain gradient of graphene increases from 5 to 20% (Fig. 3a–d), the nanocar experiences more directed motion to the unstrained end of the substrate. At lower strain gradient of substrate (e.g., strain gradient of 5%), since the driving force on the nanocar is not enough, the thermal induced stochastic motions are dominantly observed. Due to the increase of driving force at higher strain gradients (e.g., Fig. 3d), the nanocar passes the length of graphene nanoribbon more directly. According to Fig. 3a,b, the nanotruck spends a considerable amount of time moving along the $y$-axis direction; instead of moving towards the smaller potential energy region (smaller strained regions). After random movements in the directions of $x$- and $y$-axis, at the beginning of the motion, suddenly the nanocar go forth to the end of the substrate. The mentioned random movements of nanocar is attributed to the smaller acceleration of nanomachine at the strain gradients of 5% and 10%. Since the driving force on the nanocar is smaller at lower strain gradients, the nanocar motion is more impacted by the thermal fluctuations. It is necessary to remind that, the simulations are performed in the canonical ensemble (NVT ensemble). To better understand the change of the nanocar acceleration by the strain gradient, the $x$- and $y$-components of the nanocar position are illustrated as a function of time (Fig. S1 of the Supplementary Information). According to Fig.S1, by increasing the strain gradient of graphene substrate, the nanocar travels the length of substrate faster. On the other hand, due to the smaller acceleration of nanocar at the strain gradients of 5% and 10%, the nanocar experiences more random movements in the directions of $x$- and $y$-axis during the simulation.

**Figure 4:**
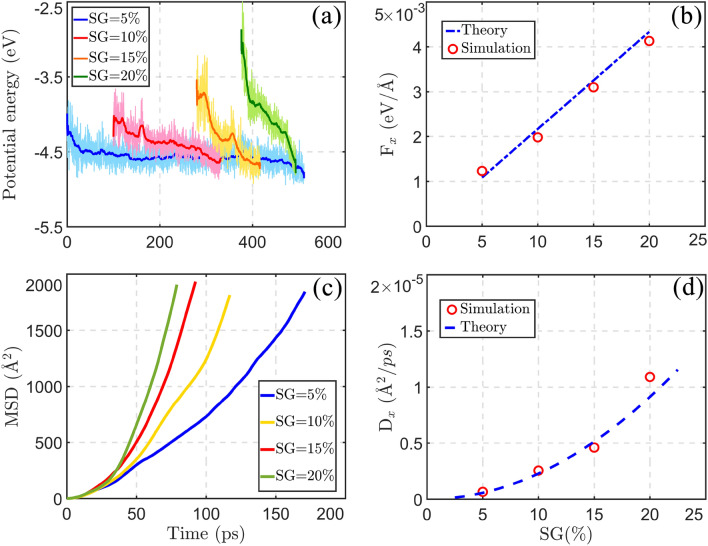
(**a**) The potential energy of the interactions between nanotruck and graphene surface. (**b**) The net driving forces, and (**d**) the diffusion coefficients of the nanotruck at different strain gradients of substrate, from theoretical and computational approaches. (**c**) The mean square displacement of the nanocar is computed from the simulations as a function of time.

To better understand the increase of driving force with the growth of strain gradient, we calculated the potential energy of the interaction of nanocar and graphene surface. As previously mentioned, the areal density of substrate’s atoms decreases with the increase of strain. When the nanocar is located on the maximum strain area of substrate, it interacts with smaller number of graphene’s atoms. On the other hand, at unstrained part of the graphene, the nanotruck has stronger interaction with the substrate, due to the increase of areal density of atoms. According to the potential energy of the interactions between nanocar and substrate (Fig. 4a), the potential energy decreases as the nanomachine travels the length of graphene layer. The decrease of the potential energy during the nanocars motion is related to the stronger interaction of nanomachine with unstrained part of the graphene. As we observe in Fig. 4a, by increasing the strain gradient from 5% to 20%, the intermolecular potential energy finds higher values at the beginning of the motion. The higher values of initial potential energy at higher strain gradient approves the decrease of intermolecular interactions with the increase of strain. Consequently, the increase of the strain gradient of substrate leads to the increase of potential energy gradient, meaning that higher driving force is acting on the nanocar at higher strain gradients.

The net driving force which is induced by the strain gradient of substrate can be calculated from theoretical approach. The potential energy between an adsorbed atom and graphene is obtained by integrating the Lennard-Jones potential energy over all atoms of the surface,

2 $$U_{adatom} = \smallint 4\varepsilon \left( {\frac{{\sigma^{12} }}{{r^{12} }} - \frac{{\sigma^{6} }}{{r^{6} }}} \right)\rho_{A} dA .$$

In Eq. (2), the $\sigma$ and $\varepsilon$ are the LJ potential parameters and ${\rho }_{A}$ is considered the areal density of substrate’s atoms. Areal density of the atoms of strained graphene is defined as,

3 $$\rho_{A} = \frac{{\rho_{0} }}{{1 + s_{x} + s_{y} }} = \frac{{\rho_{0} }}{{1 + \left( {1 - \nu } \right)s_{y} }}.$$

In this equation, ${\rho }_{0}$ indicates the areal density of the atoms of unstrained graphene and $\nu$ represents the Poisson ratio. By substituting the areal density of graphene’s atoms in Eq. (2), the potential energy between adsorbed atom and graphene surface (${U}_{adatom}$) is obtained. In this integration, the substrate is assumed to be infinite in $x$ and $y$ directions. Since the substrate dimensions are considerably larger than cut-off radius, this approximation seems appropriate. Considering the relation between potential energy and force (${F}_{adatom}=-\frac{\partial {U}_{adatom}}{\partial x}$), we find the driving force acting on adsorbed atom as,

4 $$F_{adatom} = - 4\pi \varepsilon \rho_{0} \left( {1 - \nu } \right)\left( {\frac{{\sigma^{12} }}{{5z^{10} }} - \frac{{\sigma^{6} }}{{2z^{4} }}} \right)\frac{{\partial s_{y} }}{\partial x}.$$

The detailed derivation of Eq. (4) is observable in Sect. S2 of Supplementary Information. The recent equation of the driving force on the adsorbed atom depends on the adatom height to the surface ($z$) and the strain gradient of substrate $\left( {\frac{{\partial S_{y} }}{\partial x}} \right)$. As we clarified in the computational methods, the strain of substrate linearly decreases along the $x$-axis direction. Consequently, the strain gradient term in Eq. (4) has a constant value. The net driving force on the nanocar is obtained, by calculating the sum of Eq. (4), for each atom of the nanocar. It should be mentioned that, the Lennard–Jones parameters ($\varepsilon$ and $\sigma$) are similar to those explained in Table 1. The areal density of atoms on unstrained substrate (${\rho }_{0}$) is assumed as 0.382 1/Å^2^^52^, and the Poisson ratio of graphene is 0.186^53^. Moreover, the height of the atoms ($z$) is obtained from the equilibrated position of nanocar relative to the surface. The driving force is achieved from the simulations as well, by calculating the average force on the nanocar in the direction of $x$-axis, over the simulation time. Figure 4b compares the net driving forces on the nanomachine from theoretical and numerical simulation aspects. The forces estimated by the simulations confirm the linear relation between the driving force and strain gradient of substrate (Eq. 4).

**Table 1:** Lennard–Jones parameters employed in our simulations^45–48^.

Pair atoms	$\varepsilon \left( {{\text{meV}}} \right)$	*σ* (Å)
H–H	1.45	2.65
H-C	1.34	2.81
H-N	1.97	3.03
C–C	2.41	3.40
C-N	2.50	3.41
N–N	2.60	3.42

Since we apply constant strain gradient on the substrates $\left( {\frac{{\partial S_{y} }}{\partial x}} \right)$, the driving force on the nanocar is almost constant, assuming that the nanocar height to the surface does not change significantly. As a result, the mean square displacement (MSD) of the nanotruck can be calculated from the Newton’s second law of motion. On the other hand, the MSD of nanocar depends on diffusion coefficient ($D$) by a power-law relation^54^.

5 $$MSD = {\langle x^{2} \left( t \right) \rangle} = \left( {\frac{{F_{x}^{2} }}{{4m^{2} }}} \right)t^{4} = 2Dt^{\alpha }$$

where the nanotruck coordinate in $x$-axis direction at time $t$ is shown by $x\left(t\right)$, $m$ is the mass of nanotruck, and the anomaly parameter of the motion is indicated by $\alpha$. According to the theoretical relation (Eq. 5), the MSD of nanotruck shows the super-diffusive regime of the motion ($\alpha >1$). The mean square displacements computed from the simulations approves the super diffusion of nanotruck on the graphene surface (Fig. 4c). In the next step, the diffusion coefficients of the motion were extracted from the simulations by fitting the MSD curves (Fig. 4c) with the quartic functions. As we observe in Fig. 4d, the diffusion coefficients calculated from the simulations are consistent with those obtained from the theoretical relation. Based on the employed theory, the diffusion coefficient of the motion is expected to be proportional to the second power of strain gradient.

We address another equation which may arise: Do nanocars move in the direction of their chassis? In the conventional vehicles, there is a nonholonomic constraint on the motion of cars, meaning that, when the steering angle is zero, the vehicles move in the direction of their chassis. To investigate this feature in the nano-scale cars, we achieved the direction of the chassis during the motion. In Supplementary Information, Fig. S2 illustrates the direction of the nanocar’s chassis during motion on graphene layers with different strain gradients. The results of the simulations reveal that, there is not remarkable correlation between the strain gradient of substrate and the chassis direction. The chassis vector is not in the direction of the motion at most of the simulation time. As a result, there is no nonholonomic constraint on the motion of nanocars on the strained graphene surfaces. The discrepancy between the directions of the chassis and the motion likely refers to the sliding movements of nanocar, and the minor wheel rolling motions. Akimov et al.^10^ investigated the motion of similar nanocar on gold surface, when it is exposed to the electric field. According to the results of this study, the wheel rolling mechanism is activated in the nanocar in the presence of external electric field, due to the efficient charge transfer between fullerene wheels and gold surface. The rolling movement of wheels leads to the unidirectional motion of the nanocar. It is important to remind that, in our investigation the nanocar motion is affected by the strain gradient field of substrate and the thermal fluctuations, and no external electric field has been employed. The previous studies on the motion of nanocars reported that, there is an energy barrier against the rotation of wheels around their axles (ca. 0.3 eV)^9^. The motion of nanocar is in the direction of the chassis, when this energy barrier is provided, and the rotation of the wheels should also occur synchronously. The previous investigation on the thermally activated motion of similar nanocar on the hexagonal boron-nitride surface revealed that, there is no remarkable correlation between the rolling movements of the four wheels^7^.

### Changing the temperature and strain average

**Figure 5:**
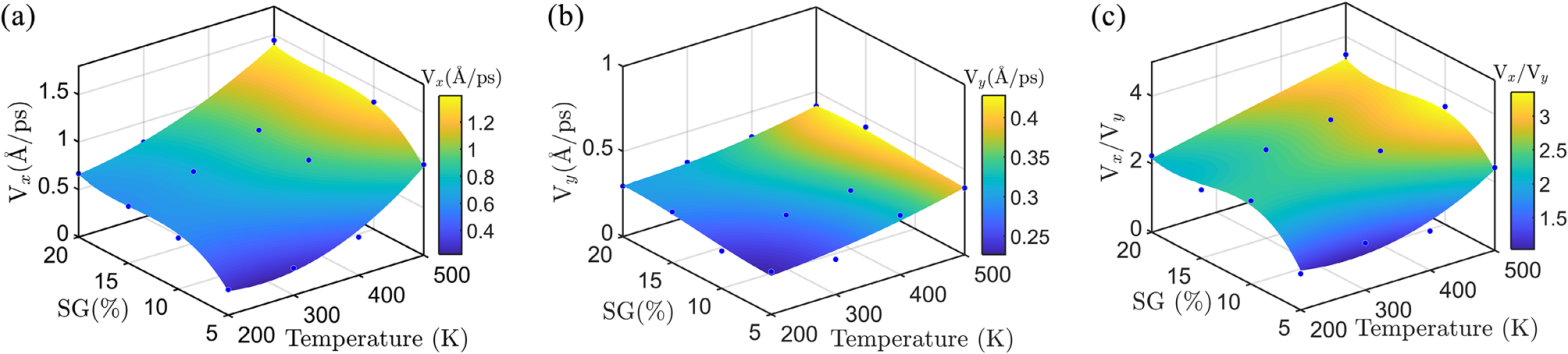
The root mean square velocities of nanotruck in the directions of (**a**) $x$-axis, (**b**) $y$-axis and (**c**) their ratio, at various temperatures and strain gradients of substrate.

In the previous section, we observed the directional motion of nanotruck on the strain gradient surfaces, at the temperature of 300 K. In this section, we first examine the directionality of the motion at various temperatures. Figure 5 illustrates the root mean square (RMS) velocities of nanocar at the temperature range of 200–500 K, and at the strain gradients of 5–20%. As it is expected from the theoretical relations (Sect. “Computational and theoretical aspects of directed motion”), at each temperature, the increase of the strain gradient of substrates leads to the increase of the velocity in the direction of $x$-axis (Fig. 5a). The growth of the $x$-component velocity with the increase of strain gradient is due to the rise of net driving force on the nanotruck. The velocity of nanocar in the direction of $x$-axis (Fig. 5a) also increases as a response to the increase of temperature. At higher temperatures, the nanocar receives higher thermal energy, thus we observe the growth of ${v}_{x}$ with the temperature. The increase of thermal energy of nanocar with the temperature is also observable in the variations of ${v}_{y}$ (Fig. 5b). It is worth mentioning that, the strain gradient of substrate which is applied along the length of graphene layer ($x$-axis), does not significantly change the velocity of nanomachine in the direction of $y$-axis.

In the next step, we calculated the ratio of ${v}_{x}/{v}_{y}$ as a criteria for understanding the directionality of the motion. In case of the rectilinear motion of nanotruck, we expect this ratio to find large values. According to Fig. 5c, at each temperature, the nanotruck experiences more directed motions (higher ${v}_{x}/{v}_{y}$) when the strain gradient increases, which is because of the increase of the driving force acting on the nanocar. As the strain gradient of graphene layer varies from 5 to 20%, the ${v}_{x}/{v}_{y}$ parameter changes by 1.17 at the temperature of 200 K, while it increases by 1.04 at 500 K. As a result, the directionality of the motion intensifies more with the growth of strain gradient at lower temperatures (e.g., 200 K), compared with higher temperatures such as 500 K.

**Figure 6:**
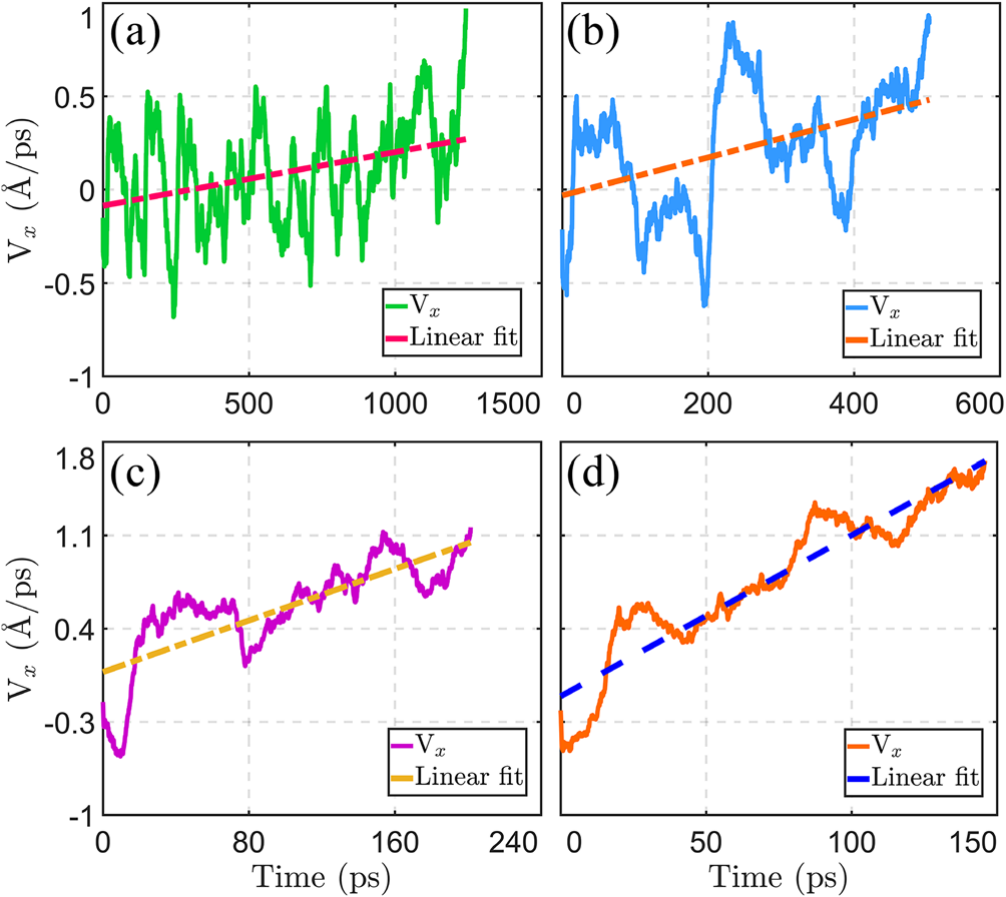
The $x$-component of the velocity of nanocar at the strain gradient of 5%, and at the temperatures of (**a**) 200 and (**b**) 400 K. The nanocar velocity in $x$-axis direction is also demonstrated at the strain gradient of 20%, and at the temperatures of (**c**) 200 and (**d**) 400 K. The linear equations are fitted to the velocity diagrams.

According to the theoretical relation of the strain-induced force (${f}_{x}$), in Sect. “Computational and theoretical aspects of directed motion”, the $x$-component of the nanocar velocity is expected to increase almost linearly with time. However, due to the thermal equilibrium of the simulation system, we expect to observe fluctuations in the velocity variation. Figure 6a,b indicate the $x$-component of the nanocar velocity, at the strain gradient of 5%, and at the temperatures of 200 K and 400 K, respectively. The velocity of the nanocar at the strain gradient of 20% and at the temperatures of 200 K and 400 K, are also depicted in Fig. 6c,d, respectively. The velocity of nanocar in the direction of $x$-axis, approximately shows linear relation with simulation time; which is better observable at the strain gradient of 20% (Fig. 6c,d). Since the driving force on the nanocar is smaller at the strain gradient of 5% (Fig. 6a,b), the slope of diagram is smaller compared to the strain gradient of 20%. Moreover, we observe larger thermal fluctuations at the strain gradient of 5%. The effect of temperature is also noticeable in Fig. 6. By increasing the temperature from 200 to 400 K, the slope of the velocity diagram increases. This is due to the increase of thermal energy, which is shared in the translational kinetic energy of the nanocar.

The rotational motion of nanotruck is studied around the axis perpendicular to the surface (Fig. S3 in Supplementary Information). The rotational angle of the nanocar (yaw angle) was obtained at different temperatures and strain gradients of substrate. Considering the normal rotational diffusion of nanocar, the rotational diffusion coefficients were calculated in the next step. In Sect. S3 of Supplementary Information, we explain the method employed to calculate the rotational diffusion coefficients. According to Fig. S3, the rotational diffusion coefficients are more influenced by the strain gradient of substrate. As we observed in the analysis of trajectories (Fig. 3), the nanocar shows diffusive motion at 5% strain gradient, while at the strain gradient of 20%, the nanocar almost indicates a directed motion. The stochastic motion of the nanocar at lower strain gradients permits the nanocar to change the direction of the motion, and experiences vertical rotations on the surface. On the other hand, at high strain gradients (e.g., 20%), the rapid movements of nanocar along the length of substrate does not allow the nanomachine to change the direction and rotate vertically on the graphene substrate. As a result, the rotational diffusion coefficient of nanocar diminishes with the increase of the strain gradient of graphene layer.

**Figure 7:**
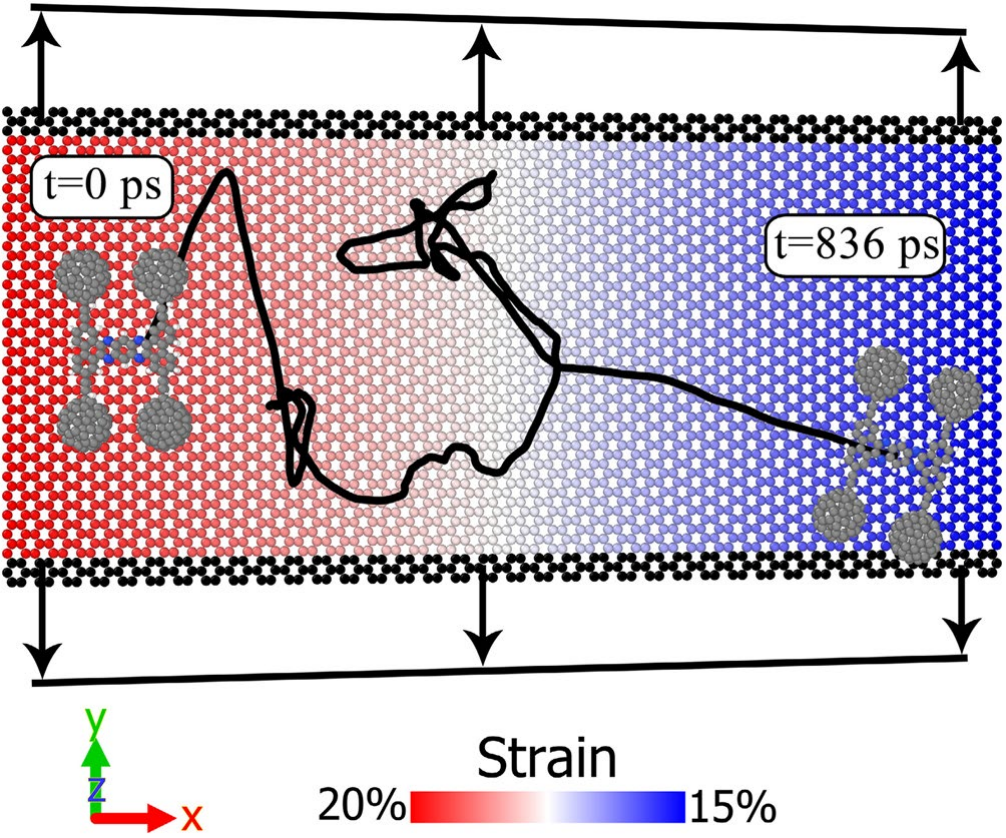
The strain of graphene layer decreases linearly, from 20 to 15%, along the $x$-axis. The strain gradient of substrate is 5%. The trajectory of the motion of nanotruck is also demonstrated in the figure, with the initial and final simulation time.

In the previous section, the strain of substrate decreases linearly, from a maximum value to zero, along the $x$-axis (see Fig. 1). In the graphene layers of Sect. “Computational and theoretical aspects of directed motion”, the maximum strain is applied to one end of the layer (i.e. $x=-75$Å), while, as we reach the other end of the substrate (i.e. $x=+75$Å) the strain value is zero. In this section (Sect.  3.2), we examine other substrate which are stretched in both ends. We consider four graphene substrates, with the strain gradient of 5%. As we move along the $x$-axis (from $x=-75$Å to $x=+75$Å), the strain of these four layers changes as: $20\%\to 15\%$, $15\%\to 10\%$, $10\%\to 5\%$ and $5\%\to 0\%$. In these substrates, the strain gradient is 5%; while, the average of the strain along the $x$-axis varies. To better understand how the strain is applied, one of these graphene layers has been shown in Fig. 7. In this substrate, the strain decreases linearly from 20 to 15% along the x-axis.

Figure S4 of Supplementary Information, depicts the trajectories of nanotruck on the substrates with the strain gradient of 5% and at the temperature of 300 K. In Fig. S3a–d, the strain of the substrates changes as $0\%\to 5\%$, $5\%\to 10\%$, $10\%\to 15\%$ and $15\%\to 20\%$, respectively. At each average of strain, the simulation was repeated five times by changing the initial condition (i.e., seed number) of nanocar. Comparing Figs. S4 and 3 it is concluded that, although the strain gradient and the average strain of the substrate alter the directionality of the motion of nanocar, the change of strain gradient has more considerable effect on the directionality of motion, compared with similar variation of average strain. By changing the strain average from 2.5% to 17.5% (Fig. S4a–d), the nanotruck shows poor directed motion, while changing the strain gradient from 5 to 20% (Fig. 4a–d) leads to the significant increase of directional movements. As we observe in Fig. S4, the increase of strain average slightly diminishes the directed motion of nanotruck. At higher strain average (e.g., 17.5%), the nanocar experiences more stochastic movements compared with lower averages of strain (e.g., 2.5%). Based on the Eq. (4), the driving force on the nanotruck is proportional to the density of substrate’s atoms (${\rho }_{0}$). When the average of substrate strain increases, the areal density of atoms on the surface decreases, which causes the nanocar to find lower driving force. Since the driving force on the nanotruck decreases, we observe more random movements at higher average strains.

**Figure 8:**
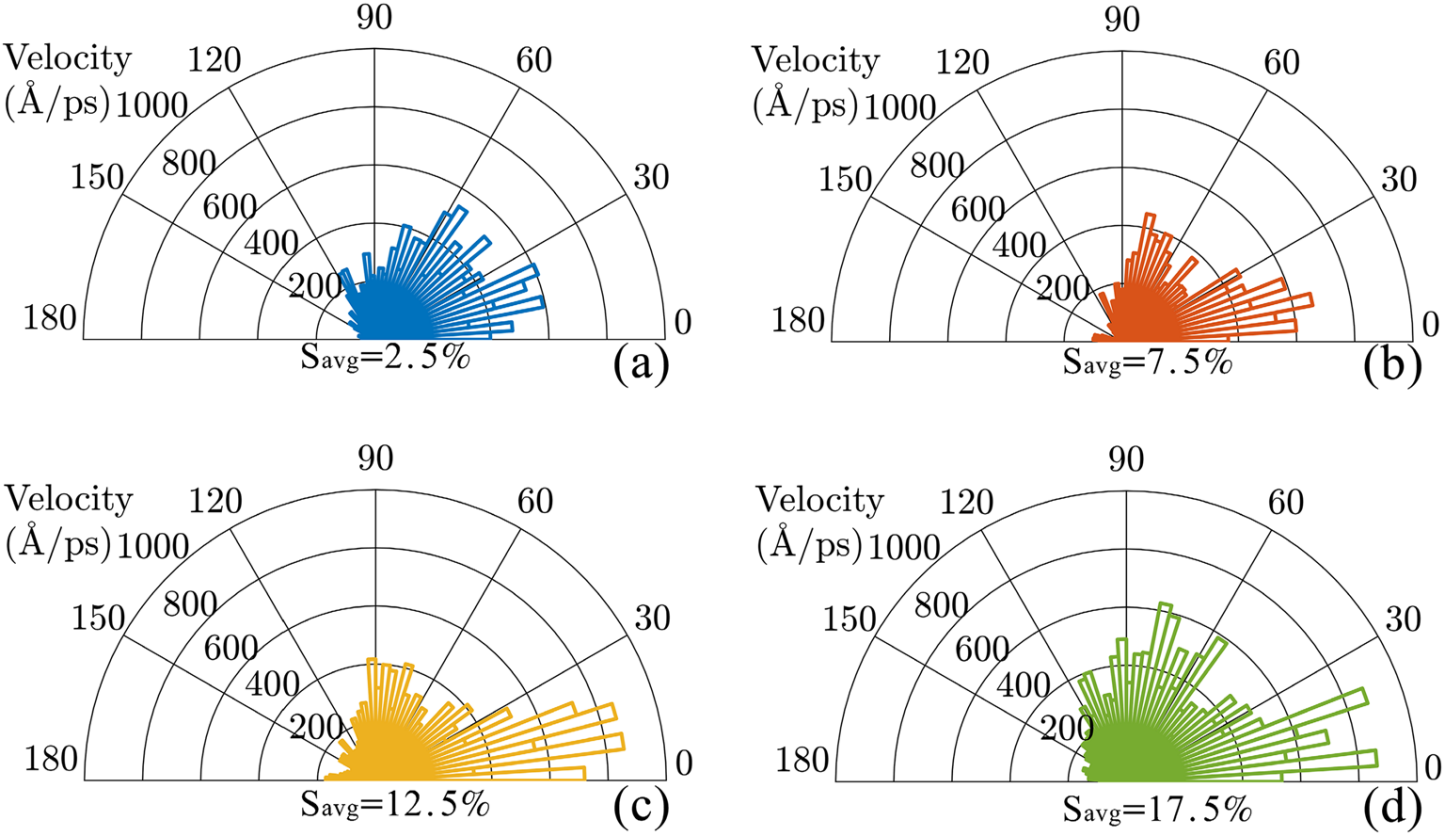
Distribution of the velocity of nanocar in different angles around the positive direction of $x$-axis. The stain gradient of all substrates is 5%, while the average of strain is (**a**) 2.5%, (**b**) 7.5%, (**c**) 12.5% and (**d**) 17.5%. The strain of substrate changes linearly along the $x$-axis.

To better measure the directed of motion at various strain averages, we analyzed the direction of velocity of nanocar. At each step of the simulation, the direction of the velocity has been measured relative to the positive direction of $x$-axis. Considering the angle between velocity and $x$-axis direction as $\varphi$, we added up the all velocities of nanocar which are in the direction of $\varphi$ angle during the steps of simulation time. To ensure the reliability of the results, the distribution of the nanocar’s velocity has been obtained for five simulations with different initial conditions of nanomachine. Figure 8 demonstrates the distribution of the velocity of nanotruck in different angles around the positive direction of $x$-axis. It should be reminded that, the strain gradient induced force is applied in the positive direction of $x$-axis. The distribution of the velocity of nanocar (Fig. 8) indicates that, the most portion of the velocities is distributed in the angles lower than 90°, which confirms the directed motion of nanocar along the length of substrate. However, by increasing the strain average from 2.5 to 17.5% (Fig. 8a–d), we observe the increase of the distribution of velocity in the angles higher than 90°, which means that the increase of strain average leads to the decrease of directional motions. This reduction of the directional motions is arising from the decrease of driving force on the nanocar, which is previously explained in the analysis of trajectories (Fig. S4).

**Figure 9:**
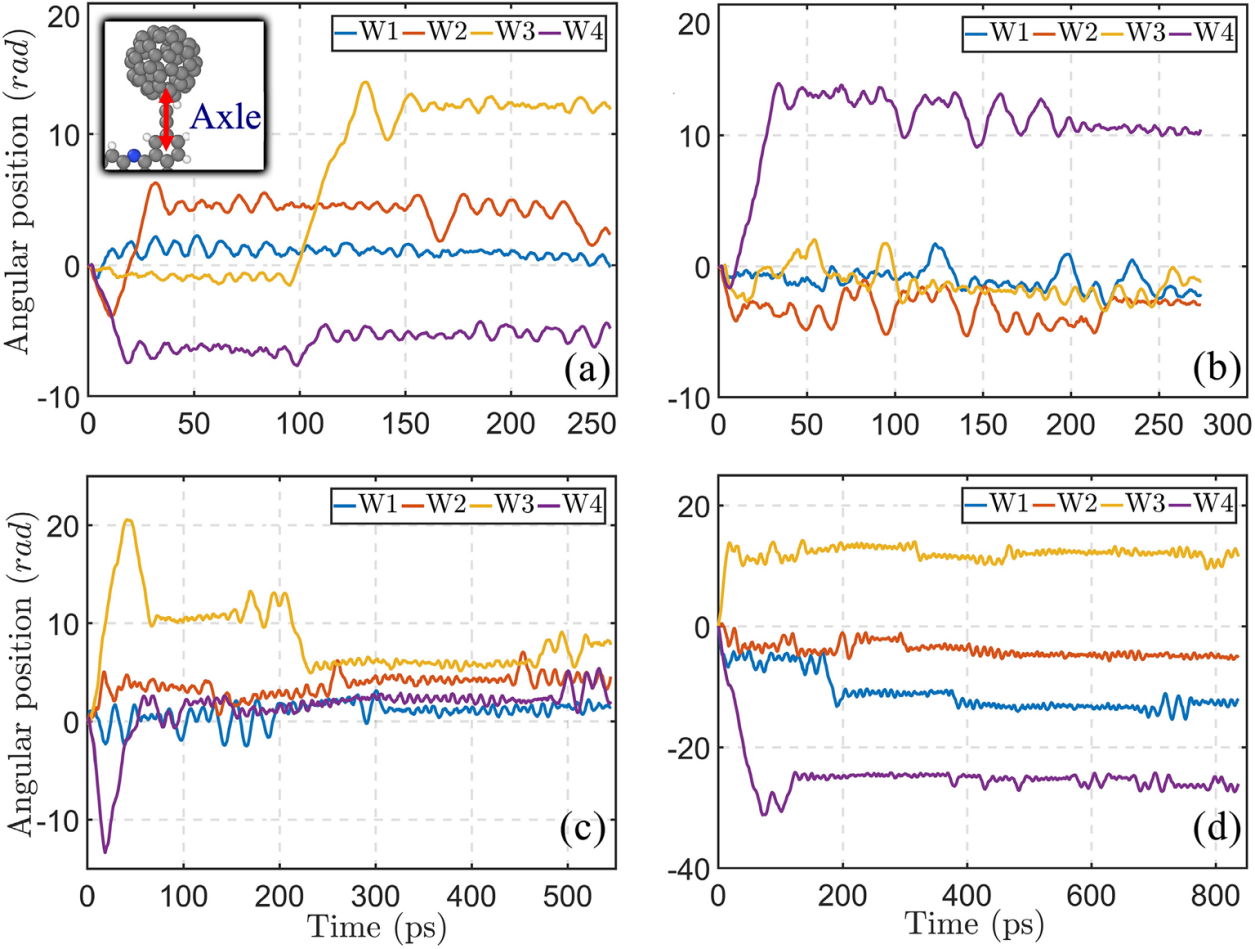
Rotation of the nanotruck’s wheels (W1, W2, W3 and W4) around their axles. The nanomachine moves on the surfaces with the same strain gradient of 5%, while the strain averages are (**a**) 2.5%, (**b**) 7.5%, (**c**) 12.5% and (**d**) 17.5%. The inset of the figure illustrates one of the axles of the nanocar’s wheels.

The wheel rolling mechanism has been evaluated, during the motion of nanocar on the graphene surfaces with the strain gradient of 5% and different average of strain. Figure 9 depicts the rotation of fullerene wheels around their axles, during the motion to the unstrained end of the graphene layer. As illustrated in Fig. 9, at different strain averages, the wheel rotation occurs rarely during motion of nanotruck. The maximum magnitude of rotation happens for the 4th wheel of nanocar (W4) at strain average of 17.5, when the angular position of the wheel reaches -31.14 radian (i.e., about 5 revolutions). As a result, the rolling motion of wheels does not constitute the majority of the mechanism of motion. It is noteworthy that, the rotations of the wheel are not synchronized, which can lead to the rotation of nanocar during motion on the surface.

### Programming the transportation of nanocar

In the previous studies, the motion of the nanotruck has been investigated on the graphene substrate^9,44,54^. In these investigations the substrate is not subjected to strain gradient, and the graphene layer has been utilized in its pristine form. According to these studies, the nanotruck has shown diffusive motion on the graphene at different temperatures. To better understand the directed motion of nanotruck in presence of strain gradient, we compared some characteristics of the motion in Table 2. As we observe in this table, the root mean square velocity of nanotruck is considerably higher on the graphene substrate with 20% strain gradient. The higher root mean square velocity at 20% strain gradient, is related to the strain induced driving force on the nanotruck, which leads to the increase of nanotrucks speed. In the presence of strain gradient, since the strain gradient is applied in the direction of $x$-axis, the $x$-component of the average velocity (${\overline{v} }_{x}$) is higher than that of $y$-component (${\overline{v} }_{y}$). The higher ${\overline{v} }_{x}$ compared with ${\overline{v} }_{y}$ refers to the directed motion of nanotruck on the substrate with strain gradient. On the other hand, the diffusive motion of nanotruck in the absence of strain gradient leads to the insignificant average velocities in the directions of $x$- and $y$-axes.

**Table 2:** Comparison of the velocity of nanotruck on graphene layers with 20% and 0% strain gradients, and at the temperature of 300 K^9,44,54^.

	$SG=20\%$	$SG=0\%$
${\overline{v} }_{x}$ (Å/ps)	0.67	0.01
${\overline{v} }_{y}$ (Å/ps)	0.05	0.02
${v}_{rms}$ (Å/ps	0.81	0.39

Several techniques have been proposed to steer the motion of nanocars on the surface. Using the electric field of STM microscope is one of the major methods, which is implemented experimentally^55^. Due to the special chemical structure of nanomachine, the molecule starts to move when the current flows through it from the STM tip^20^. The movements of nanocar can be also attributed to the excitation of vibrational modes^56^, or to the structural changes as a response to the electron flow^19^. The temperature gradient of the surface has been also reported as a method, to control the motion of nanocars^18^. The adsorbed molecule experiences the decrease of free energy, as it moves toward the lower temperature regions^23^. In the case of present study, the nanocars exhibit directed motion on the surface, when the strain gradient field is applied to the substrate. As previously observed, the directed movement of nanocar is due to the lower potential energy of nanomachine on the less strained part of the substrate. The higher areal density of atoms in the less strained regions, leads to the stronger interaction of substrate with the nanocar, and the lower potential energy. The directed motion of nanocar has an important role in transporting material and energy on the surface. The targeted transportation of molecules on the surface can be utilized to design nano-structures on the surface, through the bottom-up strategy.

**Figure 10:**
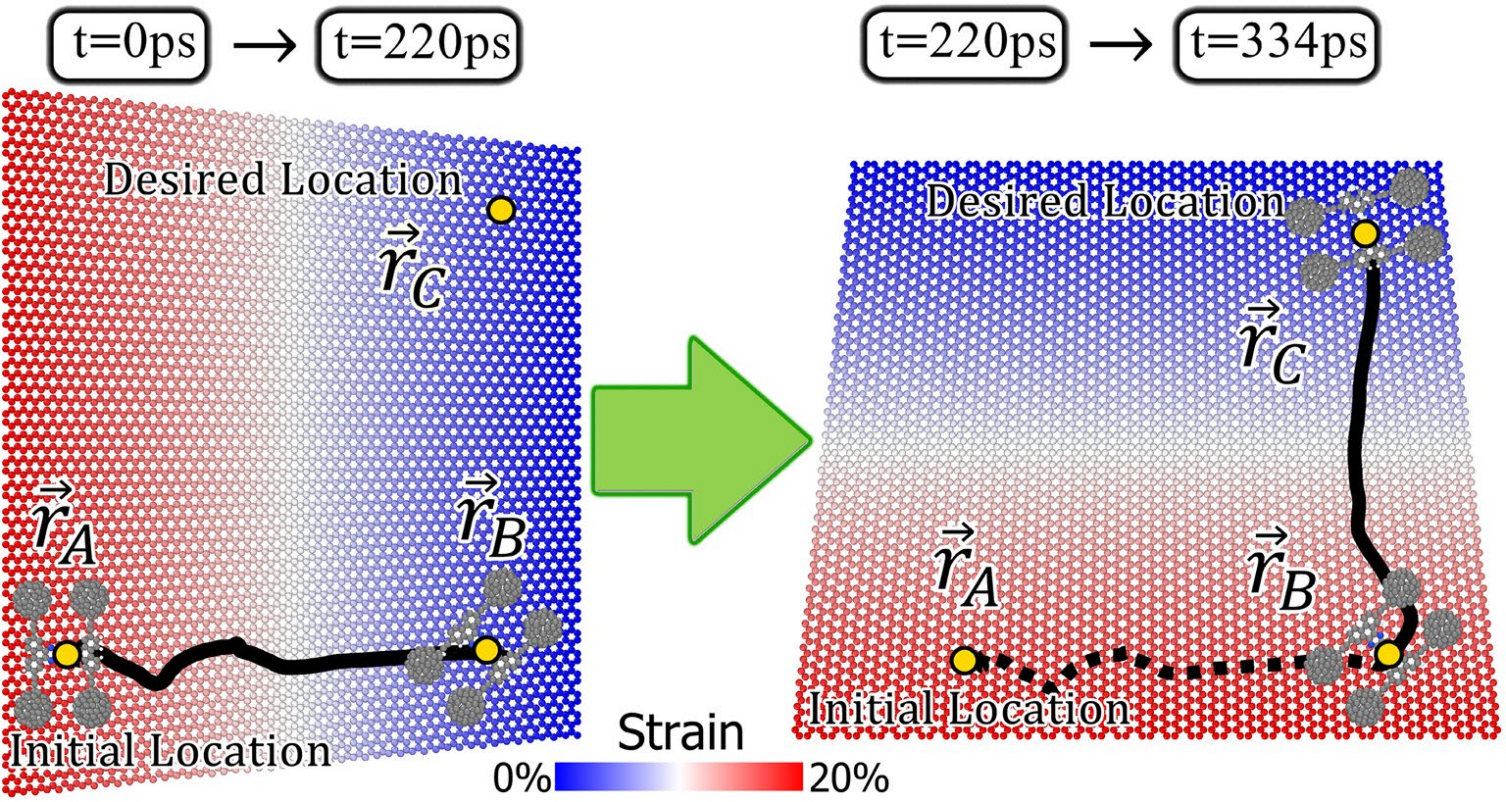
Controlling the motion of nanotruck on the graphene by applying successive strain gradients in perpendicular directions.

As previously mentioned, the nanocars have shown the potential of transporting cargoes on the surface^57^. As a result, controlling and programming the surface movements of these nanomachines are of interest. In the previous sections of this study, we proposed the strain gradient of substrate as a method to steer the motion of nanocar; and we also evaluated the appropriate condition for directed motion, from different points of view such as: the magnitude of strain gradient, the average of strain, and the temperature of the environment. Using the proper conditions, in this section, we aim to control the motion of nanotruck at the temperature of 100 K. Figure 10 indicates the steered motion of nanotruck, which is conducted by controlling the strain of graphene layer. In the first scenario, the nanomachine moves from initial position on the substrate (${\overrightarrow{r}}_{A}$) to the second point at the lower right part of the surface (${\overrightarrow{r}}_{B}$). When the nanocar reaches the desired point at 220 ps, the strain gradient is applied in perpendicular direction (second scenario). Consequently, the nanotruck starts to move in the direction of $y$-axis. In the second scenario, the nanomachine travels the width of the substrate in 114 picoseconds. According to Fig. 10, the nanocar is driven to the target point (${\overrightarrow{r}}_{C}$) at the end of the motion. Since the nanomachines are developed for carrying payloads on the surface, the steered motion of nanocars are important to design delivery systems.

## Conclusions

In the present study, we propose applying the strain gradient on substrate, as a method for controlling the surface motion of nanomachines. The simulations were carried out by molecular dynamics method. The nanomachine showed directed motion from maximum strained part to the unstrained end of the graphene nanoribbon. We observed the increase of the directionality of motion, by increasing the strain gradient on graphene substrate. The strain gradient induced driving force on the nanomachine was obtained from the theoretical point of view. The net driving force depends on the magnitude of strain gradient, areal density of substrate’s atoms, and the vertical distance of nanocar’s atoms to the surface of graphene. The results of the simulations such as, the average force on the nanocar, and diffusion coefficients of the motion are in good agreement with predictions of the employed theory.

To find the optimum condition for the directional motion, we simulated the motion of nanocar on strain gradient graphene layers, at various temperatures. By increasing the temperature from 200 to 500K, the ratio of longitudinal to the transverse speed increases, which refers to the rise of directional motions at higher temperatures. The nanomachine has also been simulated on the substrates which are similar in strain gradient, but the strain average finds different values. Considering a constant value of strain gradient on substrate, the nanocar shows more stochastic motions on the surfaces with higher average strain. The increase of random motions at higher strain average is attributed to the decrease of areal density of substrate’s atoms, which is confirmed by theoretical relations, as well.

The mechanism of the motion of nanocar was studied on the graphene surfaces with strain gradient, by computing the direction of the nanocar’s chassis and rotation of the wheels around their axles. The mentioned parameters revealed the absence of nonholonomic constraints in the motion of nanomachine. The sliding movements constitute the majority part of the motion. On the other hand, the wheels of nanocar show slight uncorrelated revolutions around their axles.

In the last step, we steered the motion of nanomachine to different sides of the graphene surface, by adjusting the strain gradients of substrate. Since the surface rolling molecular machines have shown the potential of transferring nano-payloads on the surface, controlling and programming of their movements are important for the design of delivery systems.

### Supplementary Information

Supplementary Information.

## Data Availability

The data that support the findings of this study are available on request from the corresponding author.
