# Supplementary material for: Toward steering the motion of surface rolling molecular machines by straining graphene substrate
Source: Sci Rep. 2023 Nov 27;13:20816. doi: 10.1038/s41598-023-48214-1 (PMC10682032; doi:10.1038/s41598-023-48214-1)
Supplement: Supplementary file 1 — Supplementary Information. [file 41598_2023_48214_MOESM1_ESM.docx]

# Supplementary Information

Toward Steering the Motion of Surface Rolling Molecular Machines by Straining Graphene Substrate

Mehran Vaezi^†^ and Hossein Nejat Pishkenari^∗,‡^

†Institute for Nanoscience and Nanotechnology (INST), Sharif University of Technology, Tehran, Iran

‡Mechanical Engineering Department, Sharif University of Technology, Tehran, Iran

E-mail: Nejat@sharif.edu

Phone: +98 21 6616 5543

# Table of Contents

Section S1. Supplementary figures ……………………….……………….……………...… S2

Section S2. Detailed derivation of Equation 4………………....……………….…………S4

Section S3. Calculation of rotational diffusion coefficients………….…….………...S5

Section S4. The force field parameters ………………………………………………………S6

Section S5. Diffusion regime ……………………………………………………………………..S8

# Section S1. Supplementary figures


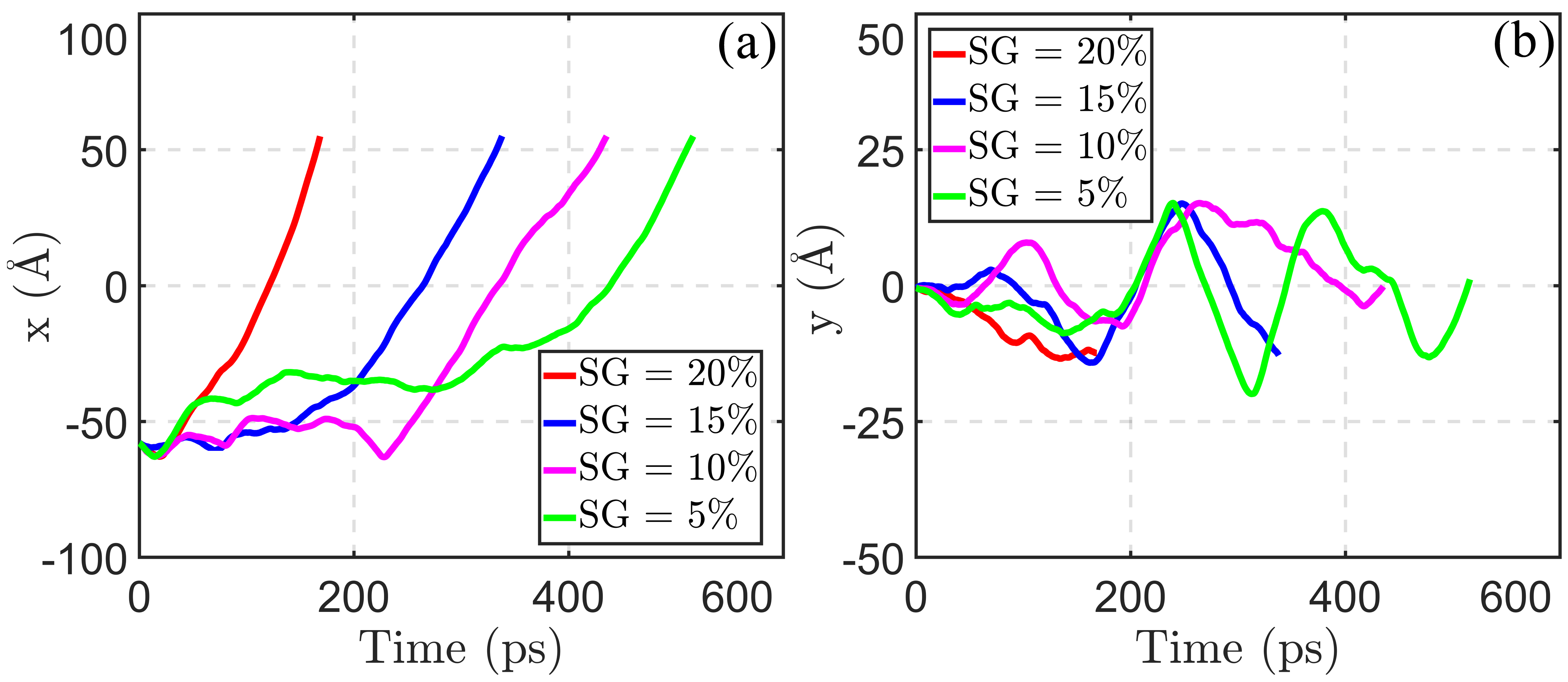


Figure S1. (a)$x$- and (b) $y$-component of the position of nanocar as a function of simulation time, and at different strain gradients of substrate.


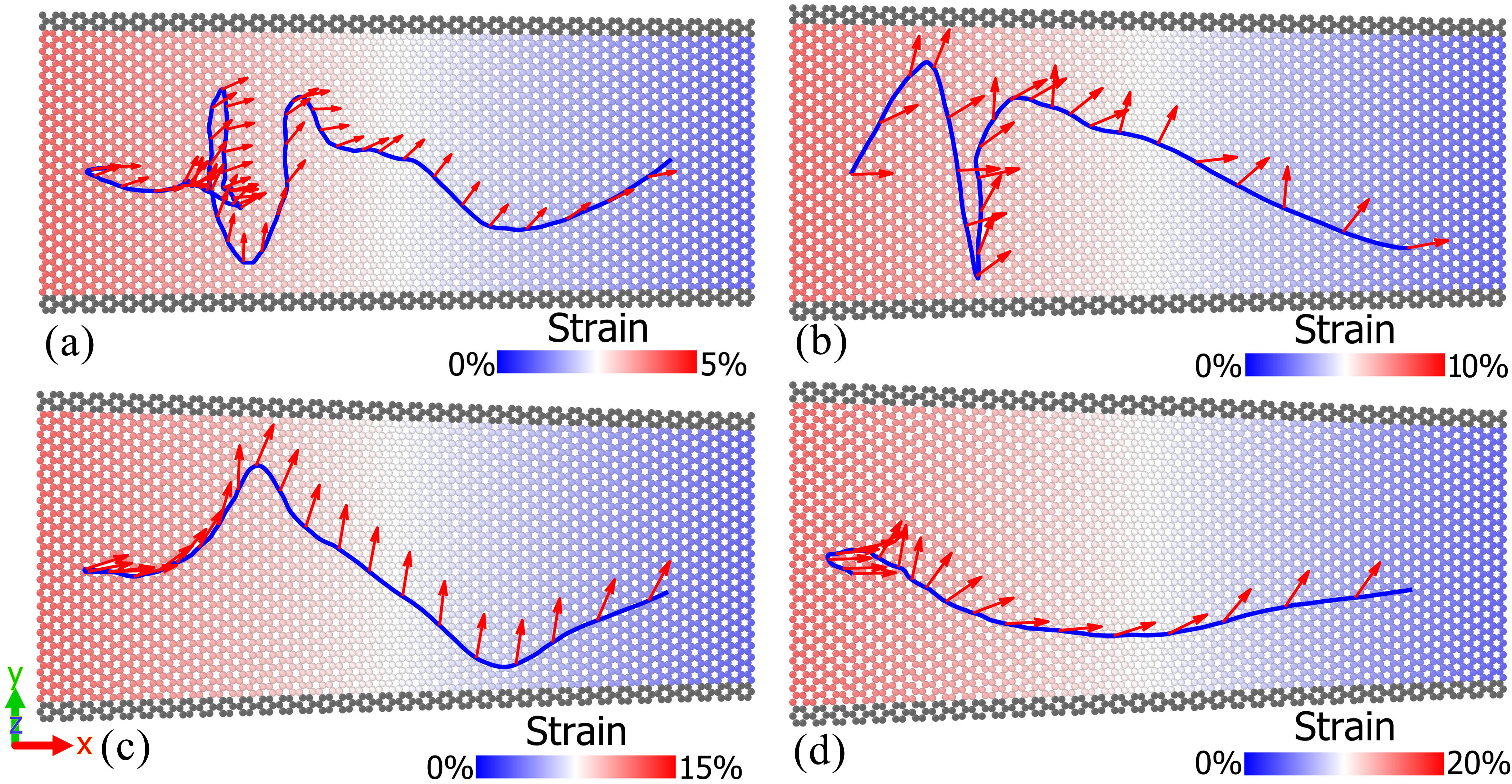


Figure S2. Trajectories (blue lines) and the direction of the chassis (red vectors) of nanotruck during the motion on the graphene surfaces with the strain gradient of (a) 5%, (b) 10%, (c) 15% and (d) 20%.


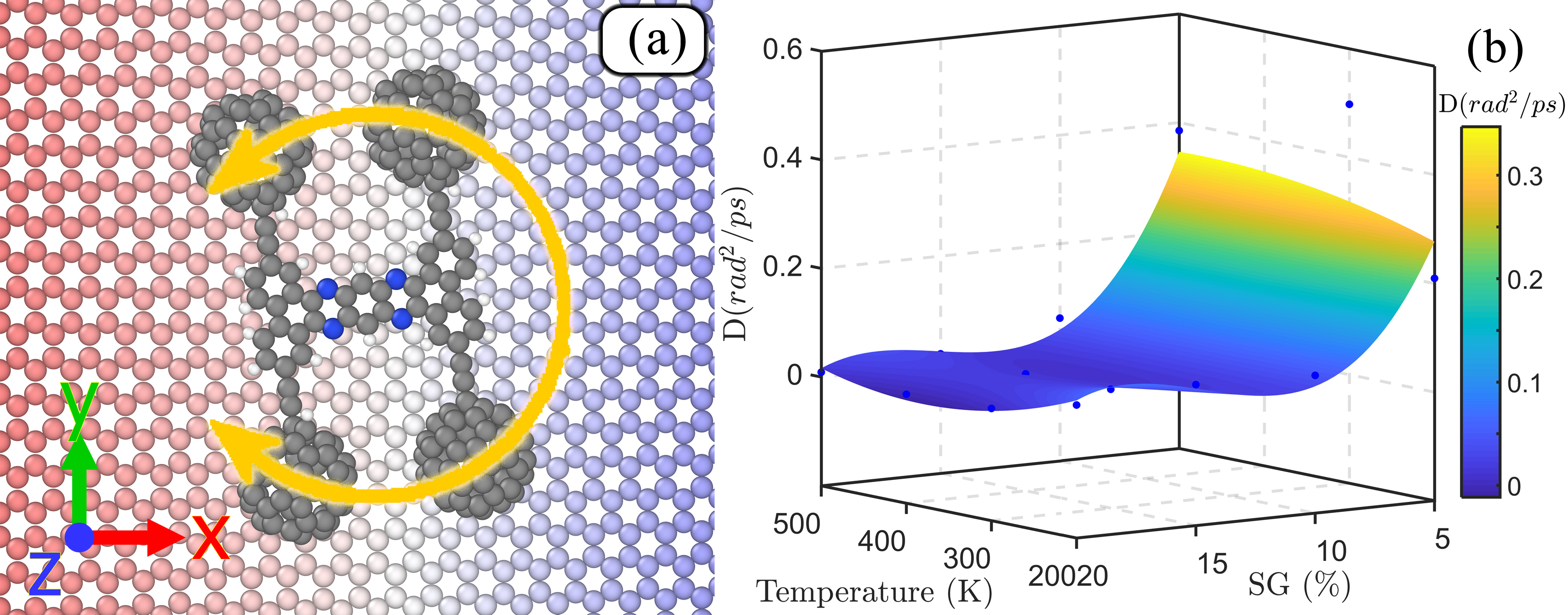


Figure S3. (a) Rotation of the nanotruck around the axis perpendicular to the surface ($z$-axis). (b) Rotational diffusion coefficients of the nanotruck at different temperatures, and strain gradients of substrate.


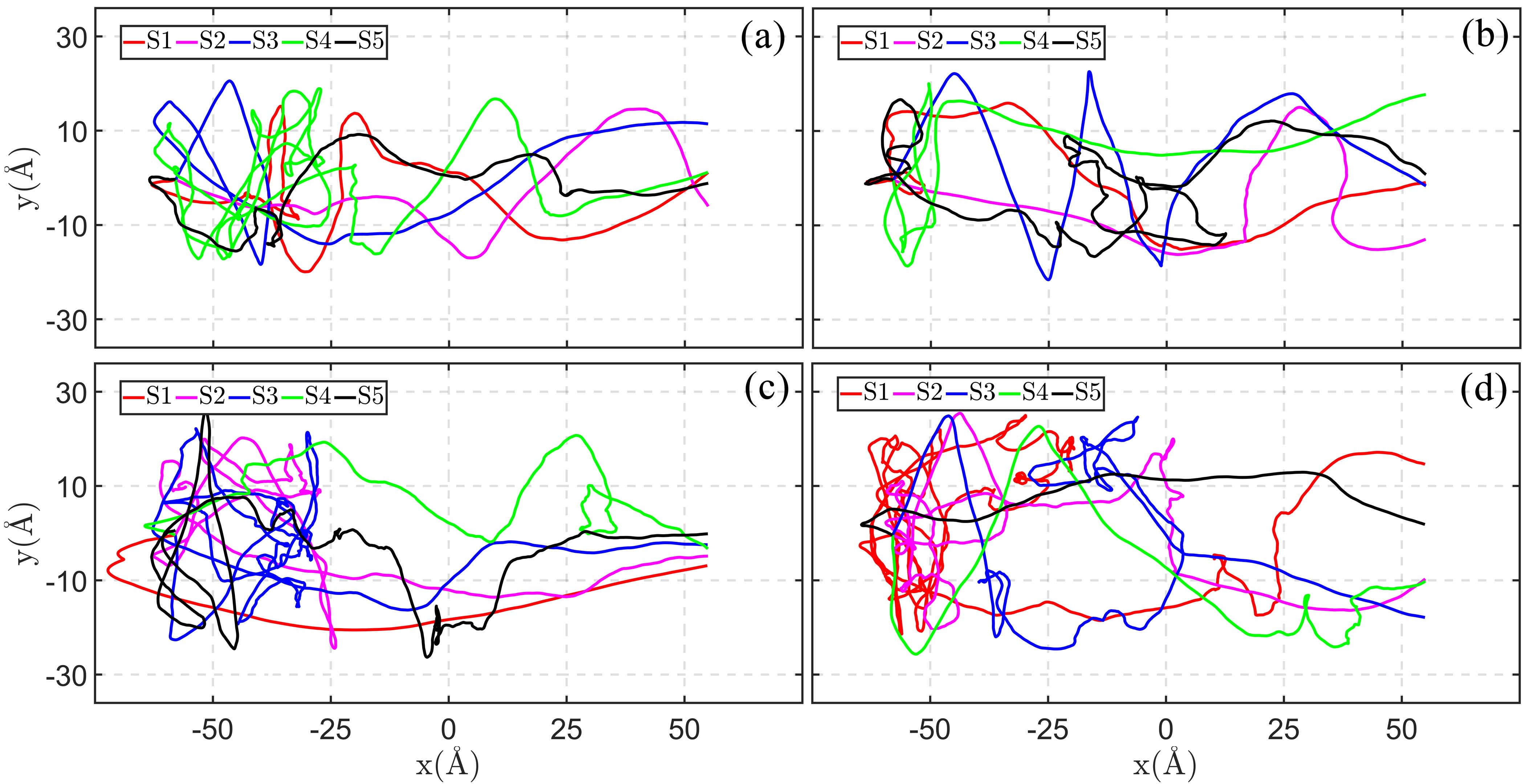


Figure S4. Trajectories of the motion of nanotruck at the strain gradient of 5%, while the average of strain is (a) 2.5%, (b) 7.5%, (c) 12.5% and (d) 17.5%. In this analysis the temperature of simulation is constant at 300 K. The strain of substrate changes linearly along the x-axis.

# Section S2. Detailed derivation of Equation 4

To obtain the net driving force on the nanotruck, we calculate the vdW interactions between an adatom and all of the atoms of graphene surface, using the Lennard-Jones potential energy as follows,

$U_{adatom}= \int4\varepsilon\left( \frac{\sigma^{12}}{r^{12}}-\frac{\sigma^{6}}{r^{6}} \right)\rho_{A}dA$. (S1)

In Equation S1, the $\sigma$ and $\varepsilon$ are the LJ potential parameters and $\rho_{A}$ is considered the areal density of substrate’s atoms. Areal density of the atoms of strained graphene is defined as,

$\rho_{A}=\frac{\rho_{0}}{1+s_{x}+s_{y}}=\frac{\rho_{0}}{1+\left( 1-\nu\right)s_{y}}$. (S2)

In the latter equation, $\rho_{0}$ indicates the areal density of the atoms of unstrained graphene and $\nu$ represents the Poisson ratio. By substituting the areal density of graphene’s atoms in Equation S1, the potential energy between adsorbed atom and graphene surface ($U_{adatom}$) is obtained.

$U_{adatom}= \iint_{-\infty}^{+\infty} 4\varepsilon\left( \frac{\sigma^{12}}{\left( x^{2}+y^{2}+z^{2} \right)^{6}}-\frac{\sigma^{6}}{\left( x^{2}+y^{2}+z^{2} \right)^{3}} \right)\frac{\rho_{0}}{1+\left( 1-\nu\right)s_{y}}dxdy$ (S3)

$=\iint_{-\infty}^{+\infty} 4\varepsilon\rho_{0}\left[ 1-\left( 1-\nu\right)s_{y} \right]\left( \frac{\sigma^{12}}{\left( x^{2}+y^{2}+z^{2} \right)^{6}}-\frac{\sigma^{6}}{\left( x^{2}+y^{2}+z^{2} \right)^{3}} \right)dxdy$

In this integration, the substrate is assumed to be infinite in $x$ and $y$ directions. Since the substrate dimensions are considerably larger than cut-off radius, this approximation seems appropriate. By calculating the recent integral in the direction of $y$-axis, the following equation is derived.

$U_{adatom}=\int_{-\infty}^{+\infty} 4\varepsilon\rho_{0}\left[ 1-\left( 1-\nu\right)s_{y} \right]\left( \frac{63\pi\sigma^{12}}{{256\left( x^{2}+z^{2} \right)}^{5.5}}-\frac{{3\pi\sigma}^{6}}{{8\left( x^{2}+z^{2} \right)}^{2.5}} \right)dx$ (S4)

Considering the relation between potential energy and force, we find the driving force acting on adsorbed atom as,

$F_{adatom}=-\frac{\partial U_{adatom}}{\partial x}=-\frac{\partial U_{adatom}}{\partial s_{y}}\frac{\partial s_{y}}{\partial x}$ (S5)

$F_{adatom}=-4\pi\varepsilon\rho_{0}(1-\nu)\frac{\partial s_{y}}{\partial x}(\frac{\sigma^{12}}{5z^{10}}-\frac{\sigma^{6}}{2z^{4}})$.

The recent equation of the driving force on the adsorbed atom depends on the adatom height to the surface ($z$) and the strain gradient of substrate ($\frac{\partial s_{y}}{\partial x}$).

# Section S3. Calculation of rotational diffusion coefficients

Considering the rotations of nanocar around the axis perpendicular to the graphene surface ($z$-axis in Figure 1 of manuscript), the rotational diffusion of nanocar is studied. Using the angular velocity of nanocar around the $z$-axis ($\omega_{z}$), one can calculate the angular position ($\varphi_{z}$) as follows.

$$\varphi_{z}\left( t \right)=\int_{t_{0}}^{t_{f}} \omega_{z}\left( t \right) dt+\varphi_{z}\left( 0 \right)$$

 (S6)

where, $t_{0}$ and $t_{f}$ are the initial and final time of the simulation, and $\omega_{z}\left( t \right)$ is the angular velocity of nanocar at the time $t$. It should be mentioned that, the initial angular position of nanocar ($\varphi_{z}\left( 0 \right)$) is assumed to be zero. Once we calculated the angular position of nanocar from the latter integration, the mean square rotational displacement is calculated as,

$MSD_{rot}= \left\langle{{(\varphi}_{z}\left( t \right)-\varphi_{z}\left( 0 \right))}^{2} \right\rangle$. (S7)

Here, the angle brackets show the ensemble averaging, which is performed by averaging the square rotational displacement over five simulations with different initial conditions (seed numbers). Considering linear relation between rotational MSD and time (i.e., normal diffusion), the rotational diffusion coefficient ($D_{rot}$) of nanocar is achieved from the Equation S8.

$MSD_{rot}= 2D_{rot}t$ (S8)

# Section S4. The force field parameters

The following set of tables contain the parameters of molecular mechanics force field, which has been employed for the modeling of nanocar ^1-3^.

Table S1. Parameters of the harmonic bonds.

| atom types | $K_{b}(ev/Å^{2})$ | $r_{0}(Å)$ |
| --- | --- | --- |
| $C2-C2$ | 48.6652 | 1.212 |
| $C2-CA$ | 30.8837 | 1.313 |
| $CA-CA$ | 25.1593 | 1.392 |
| $CA-H$ | 14.35 | 1.101 |
| $CA-NA$ | 34.596 | 1.260 |

Table S2. Parameters of the harmonic angles.

| atom types | $K_{a}(ev/\mathrm{rad}^{2})$ | $\theta_{0}(\mathrm{rad})$ |
| --- | --- | --- |
| $C2-C2-CA$ | 1.46619 | $\pi$ |
| $C2-CA-CA$ | 1.34141 | $2\pi/3$ |
| $CA-CA-CA$ | 1.34141 | $2\pi/3$ |
| $CA-CA-H$ | 1.12304 | $2\pi/3$ |
| $CA-CA-NA$ | 1.34141 | $2\pi/3$ |
| $CA-NA-CA$ | 1.34141 | 0.638$\pi$ |

Table S3. Parameters of the dihedral term.

| atom types | $K_{d1}(ev)$ | $K_{d2}(ev)$ | $K_{d3}(ev)$ | $K_{d4}(ev)$ |
| --- | --- | --- | --- | --- |
| $CA-C2-C2-CA$ | 0 | 4.34E-05 | 0 | 0 |
| $C2-C2-CA-CA$ | 0 | 4.34E-05 | 0 | 0 |
| $C2-CA-CA-CA$ | 0 | 0.650451 | 0 | 0 |
| $C2-CA-CA-H$ | 0 | 0.650451 | 0 | 0 |
| $CA-CA-CA-CA$ | -0.0403 | 0.208144 | 0 | 0 |
| $CA-CA-CA-H$ | 0 | 0.234379 | 0.046 | 0 |
| $CA-CA-CA-NA$ | 0.0433 | 0.650451 | 0 | 0 |
| $H-CA-CA-H$ | 0 | 0.390271 | 0 | 0 |
| $H-CA-CA-NA$ | 0 | 0.650451 | 0 | 0 |
| $NA-CA-CA-NA$ | 0 | 0.433634 | 0 | 0 |
| $CA-CA-NA-CA$ | 0 | 0.433634 | 0 | 0 |

Table S4. LJ potential parameters for the interactions of nanotrucks atoms.

| atom types | $\sigma$($Å$) | $\varepsilon$($eV$) |
| --- | --- | --- |
| $H-H$ | 2.672 | 0.00203 |
| $H-C2$ | 3.646 | 0.00197 |
| $H-CA$ | 3.646 | 0.00197 |
| $H-NA$ | 2.957 | 0.00220 |
| $C2-C2$ | 3.460 | 0.00191 |
| $C2-CA$ | 3.460 | 0.00191 |
| $C2-NA$ | 3.349 | 0.00213 |
| $CA-CA$ | 3.460 | 0.00191 |
| $CA-NA$ | 3.349 | 0.00213 |
| $NA-NA$ | 3.242 | 0.00238 |

# Section S5. Diffusion regime

The mean square displacements of the motion revealed that, the nanocar experiences super diffusive regime at different strain gradients of substrate. The MSDs of motion grow faster than a linear relation with time ($\alpha>1$). In the super diffusions, long steps constitute the majority portion of the displacements^4^, and the distribution of step length obeys Levy probability distribution function^5^. The distributions of step length are calculated during the simulations, at different strain gradients of substrate (Figure S5). The step lengths are defined as the displacement of the nanocar between two successive steps of the simulation. According to Figure S5, the distributions of step length are well consistent with the Levy distribution, which is due to the super diffusion of nanocar on the strained substrates.


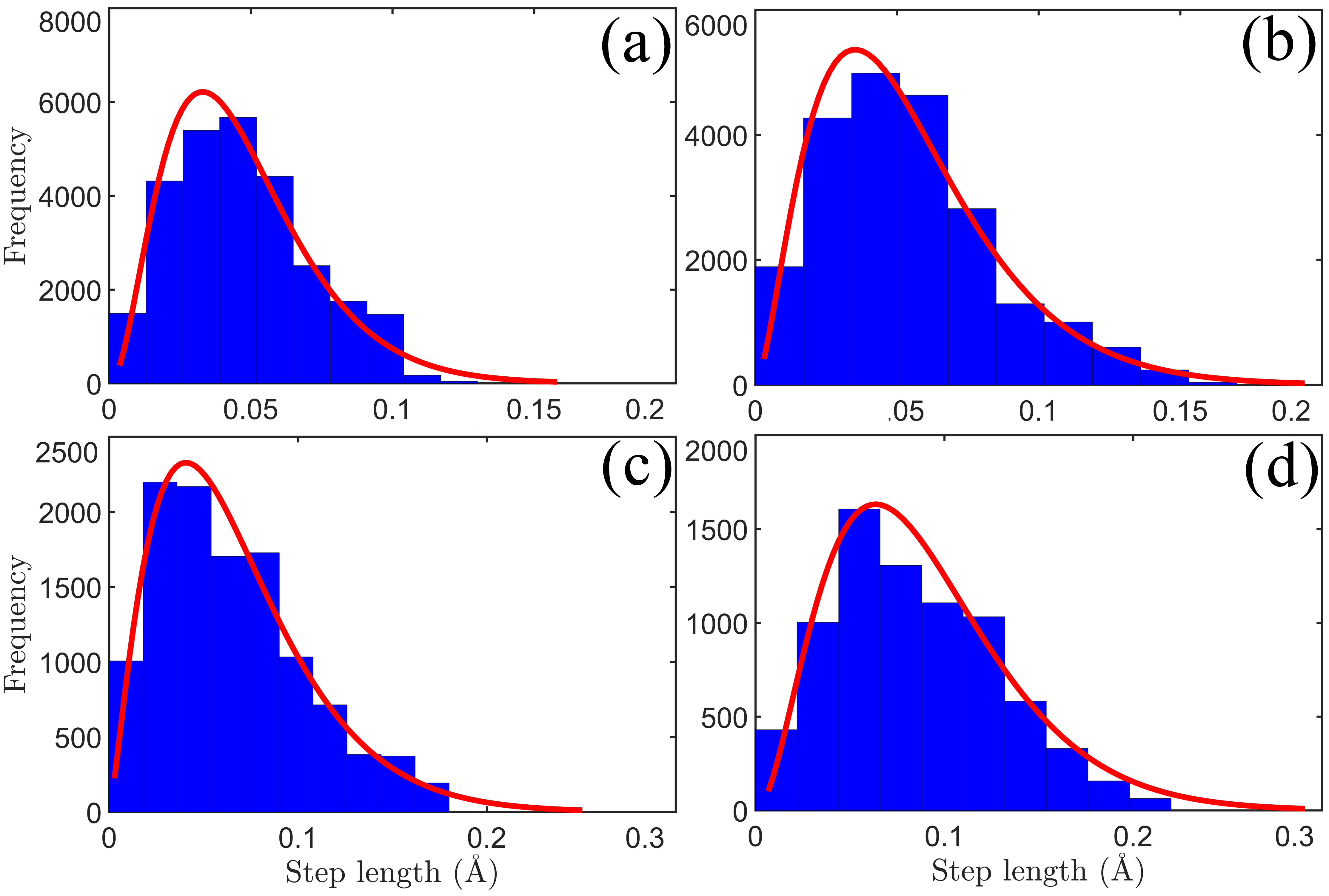


Figure S5. The distributions of step length at the strain gradients of (a) 5%, (b) 10%, (c) 15% and (d) 20%, which are consistent with Levy distribution.

# References

1. Allinger, N. L.; Chen, K.; Lii, J. H., An Improved Force Field (Mm4) for Saturated Hydrocarbons. *Journal of computational chemistry* **1996**, *17*, 642-668.

2. Allinger, N. L.; Yuh, Y. H.; Lii, J. H., Molecular Mechanics. The Mm3 Force Field for Hydrocarbons. 1. *Journal of the American Chemical Society* **1989**, *111*, 8551-8566.

3. Allinger, N. L., Conformational Analysis. 130. Mm2. A Hydrocarbon Force Field Utilizing V1 and V2 Torsional Terms. *Journal of the American Chemical Society* **1977**, *99*, 8127-8134.

4. Barthelemy, P.; Bertolotti, J.; Wiersma, D. S., A Lévy Flight for Light. *Nature* **2008**, *453*, 495-498.

5. Chechkin, A. V.; Metzler, R.; Klafter, J.; Gonchar, V. Y., Introduction to the Theory of Lévy Flights. *Anomalous transport: Foundations and applications* **2008**, 129-162.
